# A process evaluation of community-led distribution of HIV self-testing kits in rural communities in Zimbabwe

**DOI:** 10.1093/heapol/czag101

**Published:** 2026-08-10

**Authors:** Itai Kabonga, Lindiwe Mancitshana, Sharon Munhenzva, Nancy Ruhode, Mary K Tumushime, Constancia Watadzaushe, Miriam N Mutseta, Cheryl Johnson, Getrude Ncube, Karin Hatzold, Liz Corbett, Miriam Taegtmeyer, Frances M Cowan, Euphemia Sibanda

**Affiliations:** Sexual & Reproductive Health and Maternal, Newborn and Child Health, Centre for Sexual Health and HIV/AIDS Research, 10 Coronation Drive, Harare, Zimbabwe; Sexual & Reproductive Health and Maternal, Newborn and Child Health, Centre for Sexual Health and HIV/AIDS Research, 10 Coronation Drive, Harare, Zimbabwe; Sexual & Reproductive Health and Maternal, Newborn and Child Health, Centre for Sexual Health and HIV/AIDS Research, 10 Coronation Drive, Harare, Zimbabwe; Sexual & Reproductive Health and Maternal, Newborn and Child Health, Centre for Sexual Health and HIV/AIDS Research, 10 Coronation Drive, Harare, Zimbabwe; Faculty of Public Health and Policy, London School of Hygiene & Tropical Medicine, London WC1 9SH, United Kingdom; Sexual & Reproductive Health and Maternal, Newborn and Child Health, Centre for Sexual Health and HIV/AIDS Research, 10 Coronation Drive, Harare, Zimbabwe; AIDS and TB, Ministry of Health and Child Care, Harare, Zimbabwe; Global HIV, Hepatitis and STI, World Health Organization, Geneva, Switzerland; AIDS and TB, Ministry of Health and Child Care, Harare, Zimbabwe; Infectious Diseases, Population Services International, Johannesburg, South Africa; Department of Clinical Research, London School of Hygiene and Tropical Medicine, WC1E 7HT, London, United Kingdom; Department of Clinical Sciences, Liverpool School of Tropical Medicine, Liverpool L3 5QA, United Kingdom; International Public Health, Liverpool School of Tropical Medicine, Liverpool L35QA, United Kingdom; Sexual & Reproductive Health and Maternal, Newborn and Child Health, Centre for Sexual Health and HIV/AIDS Research, 10 Coronation Drive, Harare, Zimbabwe; International Public Health, Liverpool School of Tropical Medicine, Liverpool L35QA, United Kingdom

**Keywords:** community-led distribution, HIV self-testing, Undetectable = Untransmissible, distributors

## Abstract

Community-led distribution of HIV self-test (HIVST) kits has potential to combine the benefits of community-based HIV testing with the strengths of community-led interventions. We report the process evaluation from the community-led arm of a cluster-randomized trial in rural Zimbabwe comparing community-led versus community-based HIVST distribution where distributors were paid. Twenty communities were supported to design HIVST distribution models that suited their context. We conducted community meetings where we introduced community-led HIVST, including messages on the benefits of viral load suppression (Undetectable = Untransmissible; ‘U = U’). Communities determined how, and by whom, distribution would be done. Communities implemented their distribution model over 4–6 weeks. We conducted in-depth interviews with community members (*n* = 20), distributors (*n* = 20), and health workers (*n* = 20) as well as 12 focus group discussions (*n* = 91) with community members. We triangulated findings with participant observations during community meetings and implementation and descriptively analysed programme data on HIVST distribution. Qualitative data were analysed thematically. Participant observations found communities collaboratively developed and embraced HIVST distribution, demonstrating strong enthusiasm for participation. Messaging on U = U was met with interest and surprise, as many community members were unaware of this information, and became a key motivator for HIVST implementation and uptake. Across communities, 348 distributors distributed 27 812 kits door-to-door and/or from their homes or other community venues. Communities where model development was led by local leaders—rather than collective processes—expressed lower level of satisfaction and had slow implementation. Health workers supported HIVST distribution well, with opportunities for improvement noted. While distributors felt proud to deliver HIVST, many experienced logistical burdens and need for material support from the programme and community. Communities designed HIVST delivery models that were acceptable and feasible; however, efforts to make community-led models sustainable are critical. Delivering messages on U = U should continue to be prioritized and embedded within community-based HIVST distribution models.

Key messages

- Majority of countries are nearing and or have achieved the first 95 of the UNAIDS 95–95–95 HIV testing and treatment targets. Consequently, there are fewer people living with HIV who do not know their status and in need of testing, making it critical to focus on strategies such as community-led distribution of HIVST to reach people with HIV who are not yet tested and engaged in care.
- Communities collaboratively developed and implemented HIV self-test distribution models that worked for them.
- Messaging on U = U was a key motivator for acceptance of the community-led model and its implementation.
- As messaging on U = U can be understood by communities and a key motivator for implementation and uptake, it should therefore remain a priority and embedded within HIVST distribution at the community level.

## Introduction

Many countries are nearing and achieving the first 95 of the UNAIDS 95–95–95 HIV testing and treatment targets (Joint United Nations Programme on HIV/AIDS (UNAIDS) 2023). Zimbabwe, a country of HIV prevalence of 10.5% (World Health Organisation 2024) where HIV is a generalized epidemic, is one such country that has achieved the 95–95–95 targets. In such countries, there are fewer people living with HIV who are unaware of their status and in need of testing, making it critical to focus on strategies to reach people with HIV who are not yet engaged in care. In Zimbabwe and many countries in Africa, sub-optimally engaged groups include men, young people aged 15–24 years, and key population groups such as sex workers (Hatzold et al. 2019). With changes in the HIV funding landscape, there is need for implementation models that will sustainably preserve the gains made while making strides to reach the underserved groups (Baker et al. 2025, Brink et al. 2025).

HIV self-testing (HIVST) has become a critical approach for increasing access to and uptake of testing and treatment, including among those who might not otherwise be reached (World Health Organisation 2016, Hamilton et al. 2021, Indravudh et al. 2021, Neuman et al. 2021, Sibanda et al. 2021a, 2021b). Recent evidence has highlighted the role of self-testing for prevention, particularly to support pre-exposure prophylaxis (PrEP) delivery (Kiptinness et al. 2022). Community-based HIVST distribution was one of the first distribution strategies and has consistently been found to be acceptable and to achieve high testing uptake among young people, men, and first-time testers (Choko et al. 2015, Bwalya et al. 2020, Sibanda et al. 2021a). However, there was evidence that it was expensive to implement (Mangenah et al. 2019). In the context of limited and declining resources, efforts to identify low-cost and sustainable self-testing distribution approaches are essential (Sibanda et al. 2021a).

Community-led approaches are actions or strategies informed by communities, implemented by communities for communities to achieve improved health and human rights for their constituencies (UNAIDS 2022). UNAIDS and World Health Organization recognize communities as critical for HIV intervention delivery as they harness the power of community knowledge to tailor interventions to context, ensuring acceptable, appropriate, and feasible reach of priority groups (World Health Organisation 2021). There is evidence of effectiveness of community-led interventions for improving uptake of health interventions across various disease areas, including prevention of onchocerciasis and malaria (CDI Study Group 2010, Andersson et al. 2015, Roungou et al. 2015, Indravudh et al. 2019). In HIVST distribution, community-led strategies have been shown to achieve similar or better outcomes at lower cost than programmes where kits are distributed by paid distributors receiving intensive programmatic support (Sibanda et al. 2021a).

There is a dearth of knowledge of how communities can be supported to develop and implement context-relevant HIVST distribution models and of what components of a HIVST community-led model are important for success. We provide these data from an analysis that was nested within a cluster-randomized trial conducted in 40 rural Zimbabwean communities. The trial compared community-led distribution (CLD) of HIVST (intervention) with a more intensively supported community-based model where HIVST distribution was done by paid distributors. The two HIVST distribution models are described in detail below (Sibanda et al. 2021a). We found that community-led HIVST was associated with similar HIVST uptake and linkage to post-test services as distribution conducted by paid distributors (Sibanda et al. 2021a). In the evaluation survey, HIVST uptake was slightly lower in the community-led arm, 21.6%, compared with 27.5% in the paid distributor arm, adjusted OR 0.71 (95% CI 0.50–1.01; *P* = 0.06) (Sibanda et al. 2021a). The primary outcome of self-reported linkage to confirmatory testing, PrEP, and VMMC was similar between the arms, 23.9% for the paid distributor arm and 25.9% for the community-led arm, adjusted OR 1.1 (0.75–1.49). The proportion of individuals reporting a new HIV diagnosis was also similar, adjusted OR 1.1 (0.72–1.56) (Sibanda et al. 2021a). Subgroup analysis in the trial showed a trend towards better linkage to post-test services for men in the community-led model compared to men in the paid distribution arm (Sibanda et al. 2021a). In additional analyses, communities in the community-led arm who were more cohesive were better able to reach those that needed to test the most (Tumushime et al. 2025).

To inform development and implementation of community-led interventions, here we present findings of the process evaluation where we explored how and why the CLD intervention worked.

## Methods

The process evaluation was guided by the Medical Research Council (MRC) framework for designing and evaluating complex interventions (Skivington et al. 2021). As this was a process evaluation of an effectiveness trial, our perspective was a mix between the effectiveness perspective (whether intended results are produced in the real-world situation) and theory-based (to understand how and why the intervention worked).

### Initial programme theory—Undetectable = Untransmissible messaging

Prior research in the same communities had shown poor awareness of the concept of Undetectable = Untransmissible (U = U). We envisaged that U = U messaging would motivate communities to support HIVST distribution to reduce new HIV infections in their communities. Communities would collectively develop HIVST distribution models that suited their contexts and preferences and optimized uptake of HIVST among people who needed to test, while adhering to regulatory requirements.

### Description of the trial intervention

Community-led HIVST was implemented in 20 rural communities across six districts in Zimbabwe, namely, Shamva, Muzarabani, Mutoko, Zvimba, Shurugwi, and Umguza, where Population Services International (PSI) operated from. Across these six districts, two had four communities randomly allocated to the intervention arm, two had two, and the remaining two districts had five and three intervention communities each. A community was defined as a group of villages headed by a headman—a headman unit. It is an official administrative subunit through which rural community-level activities are implemented and supported in Zimbabwe. Geographic boundaries of headman units tend be based on local landscape features such as rivers, roads, and mountain ranges; hence, the sizes of headman units vary. In the 20 study communities, the headman unit sizes ranged from 4 to 36 villages, with a median of 9 villages. A village has 50–100 households.

Figure 1 summarizes how the intervention was implemented by PSI in each community. Consent for community-led HIVST distribution was obtained from the community leadership [individuals recognized for their influence, guidance, and ability to mobilize community members. In the study context, this included formal positions within local structures including chiefs (leader of a group of headman units—highest authority in the rural areas), headman (leader of a headman unit, village heads), and political leaders such as councillors] (Chigwata 2016) following detailed description of processes the community would be expected to implement and required resources. It was made clear that no financial support was available for distribution of HIVST and that communities were free to accept or refuse participation.

**Figure 1:**
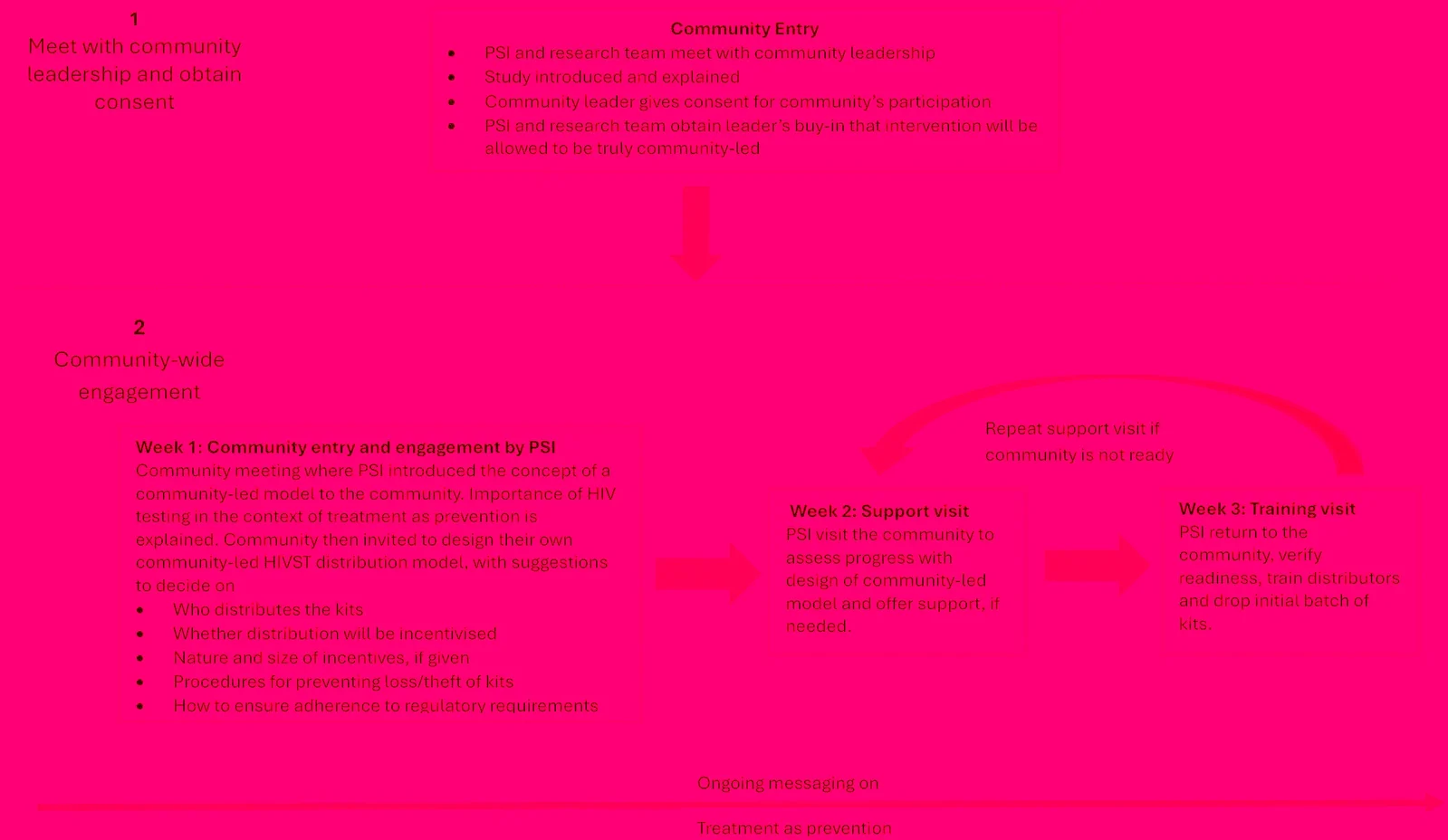
The process of engaging communities in the intervention (Sibanda et al. 2021a).

If the community leader gave consent for the community participation, they were asked to call for a community-wide meeting where PSI and the study team introduced the concept of community-led HIVST. Community-led HIVST was promoted as a strategy that could lead to reduction of new HIV infections if people living with HIV who were unaware of their status were tested and linked to treatment, based on the U = U principle (UNAIDS 2024). Communities were then asked to design context-relevant models of kit distribution that would ensure acceptability, ease of implementation, and good uptake particularly among those who might not have tested previously. They decided who would distribute kits or how kits would be accessed, what support distributors would get, procedures for preventing loss/theft of kits, and how they would ensure everyone who needed to test got an HIVST kit. They were told of regulatory standards to adhere to, including the age of consent for testing, maintaining confidentiality, and ensuring services were offered free of coercion. PSI provided flyers and posters with U = U messaging for promoting HIVST. Communities were then left to develop their distribution strategy over 1 week.

After 1 week, PSI visited the community to assess progress with the design of the community-led model and offer support as needed. If communities had developed an intervention complying with regulatory conditions above, an appointment for training of identified distributors was set for the following week. Communities not yet ready were given more time for intervention development and guidance on how they could comply with regulatory requirements.

HIVST kit distributors were trained over 3 days. The training curriculum was determined jointly between the research team and the Zimbabwean Ministry of Health and Child Care (MoHCC) and included information on the importance of HIV testing, how to use an HIVST kit, effectiveness of HIV treatment both for treating HIV and for preventing HIV infection, importance, and effectiveness of VMMC and PrEP, and how to communicate these messages. Distributors were also trained on how to support testers on relevant post-test services depending on test results. They were to inform kit recipients of the availability of post-test services from the local clinic. They were also trained on how to capture HIVST distribution data and inventory management. Post-training assessments verified distributor competence.

After training, each distributor was given a batch of 25–50 HIVST kits to start distribution in their communities, with instructions to collect additional kits from the local clinic. Distribution of kits in each community was done over 4–6 weeks between January and December 2019. People were eligible for receiving kits if they did not know their HIV status and expressed willingness to test.

### How the comparison (paid distributor) arm differed from community-led distribution arm

In the comparison arm, community leaders were engaged to identify suitable distributors—one per village. As soon as the distributors were identified, they received the same 3-day training as described above for CLD and were asked to distribute kits door-to-door in their assigned villages. [There was no model development process for the comparison arm—all communities had kits distributed door-to-door.] Distributors from the comparison arm received ongoing programme support from PSI, mainly in the form of kit replenishments from PSI field officers (i.e. they did not have to go to the clinics to complete replenishments). At the end of the distribution period, paid distributors were given a stipend of US$50. The paid distributor model was a well-polished model (Sibanda et al. 2021a) that had been previously evaluated and was found expensive owing to among other things high staff costs to maintain field presence and support.

### Process evaluation

Using the MRC framework (Skivington et al. 2021), we employed methods for understanding the context in the intervention communities and determining what was implemented and how. Data were collected between January 2019 and December 2019.

#### Understanding context

We used in-depth interviews (IDIs) with community leaders and members to understand the context in which community-led HIVST was implemented. This followed interview topic guides as described below. We also analysed programme data (collected on programme forms kept by PSI) on numbers of distributors who were trained, and we used forms completed by distributors to determine the numbers of kits distributed per community.

#### Determination of what was implemented and how

We used programme data and qualitative research to explore what was implemented and the processes followed as detailed below.

##### Programme data

Communities recorded each HIVST kit they distributed onto paper forms. Kit replenishments were made at nearby clinics; health workers confirmed forms were correctly completed before replenishment.

We analysed the programme data descriptively, computing the number of kits distributed per village and the mean number of kits distributed per distributor.

##### Qualitative research

Participant observations were conducted by trained, experienced Zimbabwean social science researchers (*n* = 5) at community gatherings and during training (i.e. weeks 1, 2, 3 meetings in Fig. 1) to capture community decisions and decision-making processes using a guide (Supplementary File S1) that documented the following:

- Who attended the meetings (composition in terms of leadership, general community, sex and age)
- Who actively participated in meetings
- Interpersonal communications during meetings
- How decisions were made, including whether they appeared to be made through consensus
- Degree to which the community appeared to have cohesion, defined as involvement and participation of everyone in the community
- Details of agreed methods of distribution

Additionally, observation visits (*n* = 120) were done in the community to explore how implementation proceeded, including whether and how promotional material was distributed/displayed and how HIVST distribution was done.

The same social science researchers conducted IDIs with community members (individuals living within the headman units) (*n* = 20), health workers in nearby clinics (*n* = 20), and distributors (*n* = 20). IDIs were conducted 3 months after distribution in Shona (local language) or English according to participant preference. IDIs were done following topic guides that explored views on community-led delivery of kits, what worked well, less well, and suggestions for improvement. IDIs also explored whether messaging on U = U reached the community and whether it was understood and how well self-testers linked to post-test prevention and treatment services. For community members, purposive sampling was done to ensure inclusion of both acceptors and decliners of HIVST kits, young and old, male and female representatives from different rural settings. We asked distributors to help identify potential participants. For distributors, purposive sampling was done to ensure inclusion of high performers and low performers (based on numbers of kits distributed—although this did not take account of the number of people who needed to test in the community). We worked with health facility managers to select diverse health workers who supported the intervention. IDIs lasted between 45 and 60 minutes.

We explored the same questions within the same timeframe in 12 focus group discussions (FGDs) (six groups for females) with community members to generate understanding of how knowledge was jointly shared/understood. FGDs lasted between 1 hour and one-and-a-half hours.

#### Analysis of qualitative data

We used the thematic analysis approach (Braun and Clarke 2006). IDIs and FGDs were audio-recorded and transcribed verbatim. Analysis started as soon as data collection began, with field notes and participant observation notes that captured both emerging descriptive and analytical themes written soon each interview/FGD/observation. Field/observation notes compared perspectives within and across participants and data collection methods, enriched through weekly team meetings that discussed theme interpretation across the social science team and informed further probes for subsequent IDI/FGDs/observations. After data collection was complete, the same team agreed on inductive coding frameworks for each category of respondents. Coding was done in QSR Nvivo, 11 (QSR International 2011).

#### Ethical considerations

The study was granted ethical approval by Medical Research Council of Zimbabwe and the authors' institutes. Written informed consent was obtained from qualitative study participants.

#### Patient and public involvement

Before the start of community-led HIVST distribution, meetings were held with communities to get views on how distribution models could be planned and implemented. In each community, we also worked closely with the community leadership including getting permission before start of activities. Communities planned and implemented their distribution models. A learning event was conducted at the end of the study where invited communities reported on their implementation of community-led HIVST and how it could be scaled in future.

## Results

### Description of the context

Study communities varied in size, intervention type, and services availability, as shown in Table 1. All 20 communities had health facilities that offered HIV testing and treatment and voluntary male medical circumcision, with PrEP available in clinics serving 14 communities. Some distributors were community health workers or had worked as community health workers before, and some had no prior involvement with health interventions.

**Table 1:** Community service availability.

Headman unit #	Availability of nearby health facilities	Health facilities offering HIV testing and treatment	Health facilities offering PrEP	Health facilities offering VMMC^a^
1	Yes	Yes	No	Yes
2	Yes	Yes	No	Yes
3	Yes	Yes	Yes	Yes
4	Yes	Yes	No	Yes
5	Yes	Yes	Yes	Yes
6	Yes	Yes	Yes	Yes
7	Yes	Yes	Yes	Yes
8	Yes	Yes	Yes	Yes
9	Yes	Yes	Yes	Yes
10	Yes	Yes	Yes	Yes
11	Yes	Yes	Yes	Yes
12	Yes	Yes	Yes	Yes
13	Yes	Yes	Yes	Yes
14	Yes	Yes	No	Yes
15	Yes	Yes	Yes	Yes
16	Yes	Yes	Yes	Yes
17	Yes	Yes	No	Yes
18	Yes	Yes	Yes	Yes
19	Yes	Yes	Yes	Yes
20	Yes	Yes	No	Yes

^a^VMMC services were offered through MoHCC and PSI outreach partnerships.

### Programme results

Overall, 348 community-led distributors were trained across the 20 communities, with 26–105 distributors per community. All 20 communities distributed kits, with 362–3 891 kits distributed per community (Table 2). All but one of 15 communities that finalized their distribution models within the suggested timelines had moderate (*n* = 8) or high (*n* = 6) involvement of communities in intervention development. Conversely, among five communities taking longer than suggested to finalize their models, two had low and three had moderate engagement, respectively (Table 2).

**Table 2:** Community distribution data.

Headman unit #	Size/number of villages	Whether met suggested timelines for model finalization^a^	Involvement of community^b^	Number of distributors trained	Number of kits distributed	Average kits per distributor	Average kits per village
1	6	Yes	Moderate	6	362	60	60
2	36	Yes	High	36	1910	52	53
3	18	Yes	Moderate	17	2018	119	112
4	6	Yes	Moderate	15	1655	110	276
5	7	No	Moderate	6	805	134	115
6	10	No	Low	19	661	35	66
7	23	No	Low	29	1393	50	61
8	12	No	Moderate	21	1645	78	137
9	10	Yes	Moderate	21	2275	103	228
10	4	Yes	Low	22	2227	117	557
11	13	No	Moderate	9	875	97	67
12	8	Yes	Moderate	17	1619	95	202
13	7	Yes	High	11	826	75	118
14	36	Yes	High	36	3891	100	108
15	4	Yes	High	12	814	69	204
16	6	Yes	Moderate	14	1042	74	174
17	12	Yes	High	26	727	28	61
18	10	Yes	High	8	1113	159	111
19	4	Yes	Moderate	7	756	95	190
20	16	Yes	Moderate	16	1195	80	75

^a^Timelines for model finalization: study teams suggested that communities finalize their models by week 2 meeting (Fig. 1).

^b^Community involvement: determined during observation in community meetings of engagement in discussions and decision-making of all categories of people: Low, decisions dominated by leadership/few key individuals; Moderate, about half of all categories of people were participating; High, everyone or all categories of people were participating.

### Qualitative study results

#### Characteristics of qualitative study participants

Among 111 community participants of IDIs and FGDs, 56 (51%) were female, and 91 (82%) accepted the offer of HIVST. Of the interviewed distributors, 12 (60%) were female, 16 (80%) were high performers, and 4 (20%) were low performers based on the number of kits distributed (Table 3). Most 16 (80%) health workers were female, with 8 (40%) primary care nurses.

**Table 3:** Characteristics of IDI and FGD community participants, distributors, and health workers.

Characteristic	Community members (*N*/%)	Distributors (*N*/%)	Health workers (*N*/%)
Age			
16–19	9 (8.1)	–	–
20–24	13 (11.7)	1 (5)	1 (5)
25–35	28 (25.2)	7 (35)	5 (25)
36–45	31 (27.9)	6 (30)	8 (40)
46+	30 (27)	6 (30)	6 (30)
Gender			
Female	56 (50.5)	12 (60)	16 (80)
Male	55 (49.5)	8 (40)	4 (20)
Marital status			
Single	17 (15.3)	–	2 (10)
Married	87 (78.4)	16 (80)	15 (75)
Widowed	4 (3.6)	3 (15)	1 (5)
Divorced	2 (1.8)	1 (5)	1 (5)
Separated	1 (0.9)	–	1 (5)
Educational level		Data not collected	Data not collected
Never attended school	1 (0.9)		
Primary	33 (29.7)		
Secondary	71 (64)		
Tertiary	6 (5.4)		
Employment status		Data not collected	
Formal	13 (11.7)		20 (100)
Informal/self employed	20 (18)		
Unemployed	76 (68.5)		
Retired	1 (0.9)		
Student on attachment	1 (0.9)		
Position in community	Data not collected		Data not collected
Village health worker		7 (35)	
Other		2 (10)	
None		11 (55)	
Number of kits distributed			
25–74	Data not required	3 (15)	Data not required
75–124		7 (35)	
125–174		5 (25)	
175–224		2 (10)	
225–274		3 (15)	
Current health care position	Data not collected	Data not collected	
Nurse in charge			5 (25)
Primary care nurse			8 (40)
Midwife			1 (5)
Nurse aide			2 (10)
Counsellor			1 (5)
Other			3 (15)
Total	111	20	20

#### Understanding how the intervention was implemented

##### Development of community-led distribution models

Across all data collection methods, we found that the development and implementation of HIVST were led by communities, as intended. In general, community members and community leaders collectively decided on how the kits were to be distributed and by whom. These decisions were made during community meetings (gatherings where community members discuss local issues, make decisions, and plan collective actions). Implemented models could be categorized into two: door-to-door delivery or mixed options with pick up of kits at distributors’ houses or other defined community places, for instance, churches, community, or shopping centres. Most communities implemented one of these models, but some implemented a combination of two or more strategies:

This program was placed in our hands as a community, so it was us who planned how it would proceed, as a community. (69-year-old, female community member, IDI)

Participant observers reported that few young people attended the intervention development meetings: ‘…young people aged between 16 and 19 were not well represented, only one (young) woman was in attendance’ [observation report (OR), community 20]. Observers also noted that intervention development, implementation, and acceptance were driven by community members who attended the week 1 visit and that in some communities, information and involvement did not cascade fully to those who did not attend.

Isolated cases were identified during community planning meetings in communities 6, 7, and 10 where community leadership exerted control over decision-making regarding the selection of distributors, catering teams (the programme offered food during distributor training and communities decided how the catering would be handled), and the distribution strategy.

In these communities where there was centralization of decision-making by leadership, communities struggled to meet timelines for finalizing the distribution strategy. Observation reports showed that where community leaders (three communities) dominated decision-making, there was discontent and general lack of interest in the intervention among community members ‘…people are not keen to participate any further because you didn't give us food at the HIV self-test introductory meeting’ (OR, community 7). In contrast, participant observers did not observe any discontent in communities where intervention development was collective.

##### Implementation of community-led distribution models

The implemented HIVST models were viewed to be effective in reaching people who are usually missed by other testing programmes. Examples given by distributors and health workers included artisanal miners (who are typically unable to leave their mining sites due to tight work schedules). Door-to-door distribution conveniently allowed access to kits by men, couples, and apostolic sect members (some apostolic sects discourage uptake of conventional health services). Distributors reported that young people preferred collecting kits from distributors’ homes to avoid being judged by parents as being sexually active:

There are artisanal miners who cannot spare some hours to walk here (to the clinic)… when just seated they feel like they are wasting time. So, when they are where they are (mining sites) I think it worked. (46-year-old, female HCW)

While there was generally strong adherence to agreed distribution models, we observed cases where distributors adapted to the local context and need. In some communities where door-to-door distribution was planned, distributors still provided kits when people visited their homes to request them. Some distributors took advantage of community gatherings, deviating from the agreed home or door-to-door distribution approach. These observations were corroborated in the IDIs and FGDs:

…they were not only targeting homes like other programs… like anyone whom they meet herding cattle, someone …in his fields, someone in his garden. So, I think it covered all areas. (37-year-old, female HCW)

Evidence from the different data collection approaches showed how distribution models which were implemented largely adhered to regulatory guidelines of limiting distribution to people who aged at least 16 years old and avoiding coercion in uptake of kits.

… It was up to you to accept or refuse, no one was forced. (34-year-old, male CM FGD)

There were sentiments from community members that the implementation of models was short-lived and should have continued to ensure convenient access to HIV testing at any time:

…they would come and say aah that time you gave us (the kits), three months have lapsed give us again…, so that we self-test again. (30-year-old, female distributor)

HIVST kits were highly acceptable and in demand. During community entry activities, many community members voiced that they wanted distribution to begin immediately and were feeling impatient about having to first go through the processes of intervention development, identification of distributors, and training, ‘Give us the kits and we will distribute them immediately’ (OR, community 19). Further evidence of this demand was demonstrated by how quickly the first batches of kits given to distributors in most communities ran out. Participant observations and IDIs found that distributors in many communities typically replenished the initial batch of kits within 1–3 days of receiving them:

At times those 25 kits I received would run out within a very, very short time. Then I would get back to the clinic to collect more. (51-year-old, male distributor)

During participant observations, there was evidence that information about the programme had spread around the community: even before distribution started, study or programme teams were frequently stopped by community members who asked for more information about the kits, mostly enquiring when the kits would be coming to the community. Observations, IDIs, and FGDs showed that word about kits spread from the community engagement meetings through word-of-mouth—encounters with distributors or people who attended the meetings. Posters and flyers are believed to have had minimal impact because observers only saw a limited display of them, despite communities having received such materials from PSI.

Evidence from the different data collection approaches showed how distribution models which were implemented largely adhered to regulatory guidelines of limiting distribution to people who aged at least 16 years old and avoiding coercion in uptake of kits.

… It was up to you to accept or refuse, no one was forced. (34-year-old, male CM FGD)

There were sentiments from community members that the implementation of models was short-lived and should have continued to ensure convenient access to HIV testing at any time:

…they would come and say aah that time you gave us (the kits), three months have lapsed give us again…, so that we self-test again. (30-year-old, female distributor)

##### Distributor workload and perceived benefits

Distributors were excited to be selected by their communities for this role as they were going to carry the tag of being ‘experts’ in HIVST, with potential to increase their popularity in the community. Additionally, distributors were also motivated by a genuine desire to serve their communities in the fight against HIV. Observers noted much eagerness and zeal among distributors during the distributor trainings:

There was a bit of excitement around being the self-testing ‘experts’ in the community going around distributing kits and giving people information about self-testing. (OR, community 20)

Observations made before implementation revealed that distributors without a health background expressed concerns about potential judgment from those with a health background, including village health workers. They feared communities were not going to listen to them nor accept HIVST kits from them compared to well-known community health workers. However, evaluation interviews with community members and distributors did not indicate any differences in the way community members viewed the distributors, indicating communities were accepting of all cadres of community volunteers.

Though distributors felt happy being selected, they expressed their work was not easy as some had to walk long distances distributing and replenishing kits with limited material support from the community. IDIs and FGDs showed that distributors were emotionally supported by communities through words of encouragement and appreciation of the good work they were doing:

… we came across elderly people…, who gave us encouragement… for us to continue with our work. (33-year-old, female distributor)

However, discussions with distributors showed they would have appreciated more material support such as food and transport to make their work easier. Community members confirmed in interviews that no material support was given to distributors by the community. Although it was reiterated during community engagement and distributor training that no financial support would be given by the programme, distributors believed they would get some token of appreciation for their work, as has become the norm for many programmes introduced in Zimbabwean communities. Distributors therefore felt they were let down by both communities and programme teams in this regard. Communities also felt the programme should have provided more support:

…they (community members) were now saying haa you are being used for nothing…it (the community) should have intervened to say, look, these young people have gone into the field, and they are not getting anything. Support them with food, cook for them when they reach your households…but that didn't happen. (51-year-old, male distributor)

The support I expected, sometimes I would be hungry when I left home, yet our community is large geographically…I wouldn't even have water to drink, yet I was supporting them so that they could test themselves and have brighter health prospects. (26-year-old, male distributor)

##### Support for community-led distribution by health facilities

Distributors were given limited HIVST stocks, with instruction to replenish from the nearest clinics. Observations and IDIs showed that distributors and health workers agreed on designated replenishment days, with clinics identifying responsible health workers for managing the replenishment process. These efforts were successful in ensuring convenient access to kits within the community. Distributors reported that they faced no significant challenges in replenishing their kits. However, a recurring theme among distributors was the long distance to replenishment centres in certain communities:

… but the challenge was that I go to a clinic which is located 12 km away from my home. So it feels like a burden, and I must ask for lunch money from my husband because it will take me the whole day to go and come back. … (52-year-old, female distributor)

As a mitigation, some communities organized themselves such that one or a few distributors would collect on behalf of others.

It was evident that knowledge of the HIVST programme was not always cascaded to all health workers. On days where the individual staff member who knew about the programme was not at the clinic, distributors faced delays with replenishment.

#### Understanding how change occurred

##### Undetectable = Untransmittable

Participant observations showed the concept of U = U was understood by community members who attended week 1 meetings and distributors attending training. Use of plays, songs, and other innovative approaches enhanced understanding of U = U during training. This capacitated distributors to disseminate the message of U = U to community members:

…. they explained clearly how one who has a virus can reduce it. Also, how they can prevent the spread of the virus between two people who have the virus. And then between a person who has the virus and one who doesn't. (41-year-old female, CM FGD)

This comprehension of U = U facilitated uptake of post-test care among those who tested positive for HIV:

They understood that if…if they got treatment and continued on the medicines, those with the virus would not pass it on to others because it would have reduced in their bodies. (41-year-old, female, CM FGD)

Of note, the concept of U = U was new to communities. Communities found this fascinating—often evoking reactions of great surprise and disbelief, followed by excitement about the potential for their communities when they understood what was being explained:

Yah, I once heard about it but… I was surprised and questioned if it was true that even if someone is HIV positive and taking their medication, if they have sex with someone will they not infect them. (24-year-old, female, CM IDI)

However, cascading this information to the rest of the community was difficult—it seemed U = U was a complex concept to understand with communities requiring additional education and explanation.

### Refined programme theory

U = U motivated communities to design models that reached people in need of HIVST. Other factors were also important for success, such as leadership motivation, community support, community cohesion, and the novelty of HIVST kits. Based on this, the refined programme theory is shown in Fig. 2.

**Figure 2:**
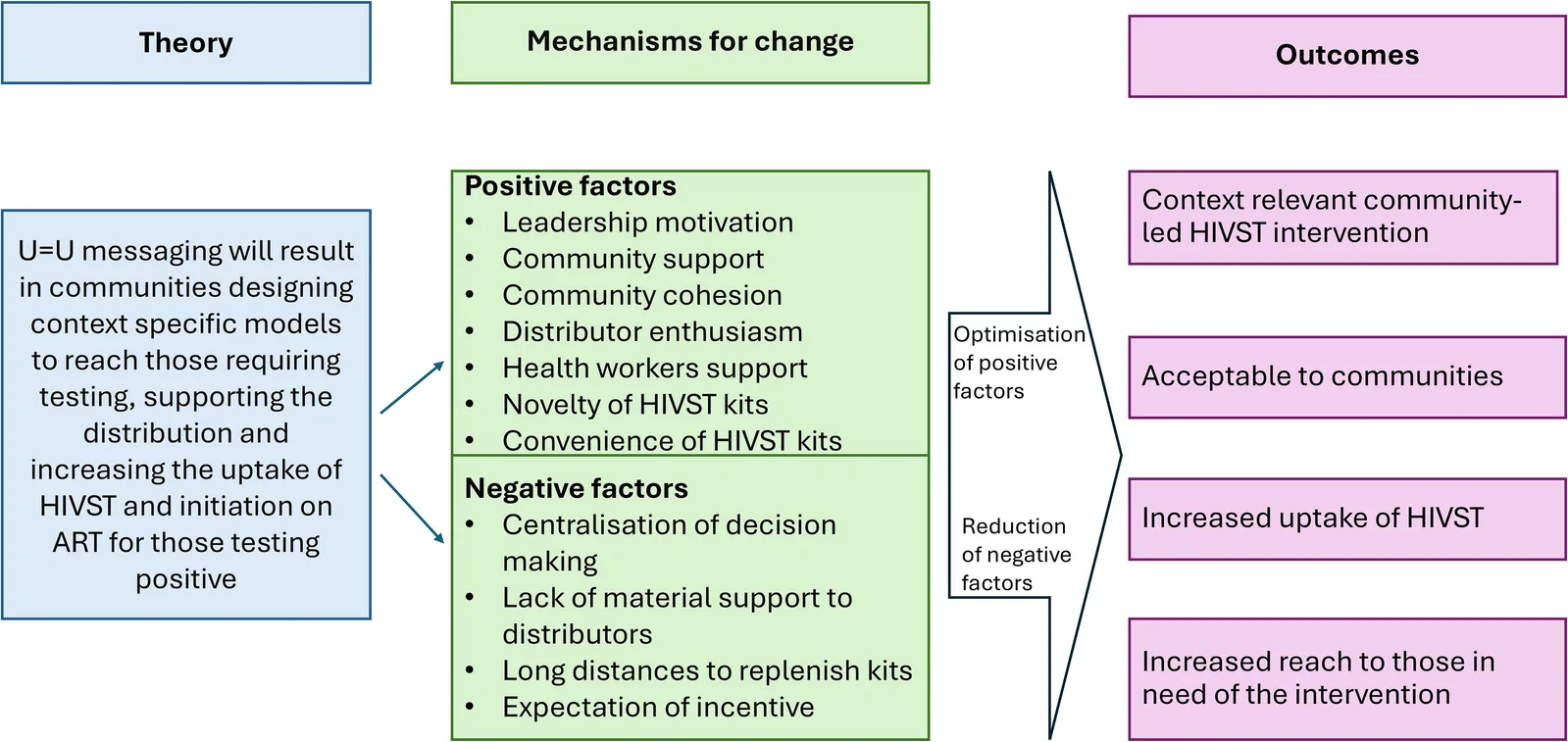
Refined programme theory.

## Discussion

The main findings from this process evaluation of the CLD arm of a cluster-randomized trial comparing two approaches to HIVST kit distribution in rural Zimbabwean communities were that communities can be supported to develop and implement context-relevant HIVST distribution models that are acceptable to communities, HIVST distributors, and health workers. Importantly, we found that messaging on U = U was understood in communities and central to driving and motivating communities to design models to reach people in most need of HIVST. Collective decision-making was key to success. Although distributors reported feeling motivated and honoured to drive the novel intervention, they reported feeling burdened in terms of time and long-distance travel and would have appreciated material support from the programme/communities. This is the first study to document detailed information on how context-relevant community-led HIVST programmes were developed and implemented, including the central role of U = U messaging on driving acceptance of the intervention.

Of note, although knowledge of the effectiveness of ART for preventing HIV transmission of HIV is not new (Cohen et al. 2011, 2012), communities were not aware of this before the beginning of the study. This is important information which can drive uptake of HIV services and reduce stigma (Paschen-Wolff et al. 2020). We showed that communities could understand the concept of U = U and distributors were able to cascade information on U = U to the communities they served. There was also evidence, however, that not everyone understood this important concept, as reported elsewhere (Prati et al. 2016, Card et al. 2018), which calls for interventions for simplifying the U = U message. The drama/plays conducted during distributor training were useful for promoting this understanding.

We added to the evidence that communities can design context-relevant models that promote intervention acceptability and uptake. When communities design models, they can tailor them to the context and needs of the community members, (Indravudh et al. 2021, Sibanda et al. 2021a), which consequently improves acceptability, access, and uptake (Rifkin 2014, Sharma et al. 2015). Community cohesion (Kawachi et al. 2008, Tumushime et al. 2025) could be important for driving development of appropriately tailored interventions. In a separate paper from the same study (Tumushime et al. 2025), we show that communities who were closely knit were more likely to reach people who self-reported a new HIV diagnosis. As HIVST was a novel intervention during the study, the intervention and its potential were not part of the community's agenda. However, the communities were aware of gaps in uptake of HIV services among them and they embraced the offer of an intervention that would address this. This highlights the importance of awareness of community needs so that programmes can suggest relevant interventions that have potential to meet these needs. As demonstrated in the three communities where the intervention was not truly community-led, where communities fail to see the importance/benefit of the intervention, it will likely fail, as discussed below.

We found HIVST distribution models where communities were more collectively involved in model development were more acceptable. This is consistent with previous studies that have provided evidence of increased coverage, efficiency, and improved health outcomes when communities lead the design, implementation, and evaluation of health interventions (Coates et al. 2014, Indravudh et al. 2021). Examples include community-led delivery of HIVST in Malawi, which was effective in increasing uptake of HIV testing among older adults, men and adolescents (Indravudh et al. 2021). This impact was driven by successful context-specific planning and trust among community members (Indravudh et al. 2019). Outside HIV, other successful examples include community-led sanitation programme that significantly reduced open defaecation in Mali (Pickering et al. 2015). Examples of projects that failed because of inadequate community engagement include a water, sanitation, and hygiene (WASH) project in Zimbabwe where communities were presented with systems they could not maintain due to exorbitant costs of replacement parts (Chinyama et al. 2021, Barrington et al. 2025). In our study, engagement of community leaders and explaining the importance of ensuring the intervention was led by the community was key to avoiding top-down approaches in most communities.

Ethical justification has been provided for greater participation and ownership of health interventions by those who are affected or those who potentially benefit from interventions. Interventions driven by communities are usually relevant, community centred, inclusive, and respectful of community interests (de Carvalho Corôa et al. 2024). However, it is important to ensure that task shifting to communities is safe and equitable and does not further burden the poorest in the society as per WHO's task shifting guidelines (World Health Organisation n.d.). Although communities were able and enthusiastic to drive HIVST kit distribution interventions, provision of additional support is important to ensure distributors, the majority of whom are women and are potentially disproportionately burdened by community-led initiatives (Ngongo et al. 2024), do not bear the burden of intervention implementation. Providing support such as transport (e.g. bicycles) and allowances for food and drink during implementation is important (Abdel-All et al. 2019, Abuya et al. 2021), and these need to be agreed upon with the community (World Health Organisation n.d.) ensuring they align with distributors level of responsibility (Glenton et al. 2013). Previous studies converge on the importance of incentives and other support in lessening the burden for community health workers (Kironde and Klaasen 2002, Mlotshwa et al. 2015, Ndu et al. 2022). Importantly, when considering the cost and cost effectiveness of community-led approaches, it is important to take the societal perspective, so intervention costs are not underestimated. Taken together with the notion that communities are not always aware of new health interventions that can benefit them, the study highlights the importance of programme support to both initiate and sustain community-led interventions.

The findings of this study come at an important time, marked by significant reductions in donor funding for the global HIV response (Brink et al. 2025). Such funding cuts threaten the availability and continuity of HIV services, particularly in low-income countries such as Zimbabwe that continue to carry a high HIV burden (Brink et al. 2025). In this context, community-led models of service delivery become increasingly important. Community-led distribution of HIV self-test kits offers a practical and sustainable approach that is less resource-intensive, less dependent on highly specialized health personnel, and potentially more cost-effective than models relying on paid distributors and facility-based models. By leveraging on existing community structures, community-led models can help maintain access to essential HIV services among underserved populations, even within constrained funding environments. The study therefore highlights the potential of community-led HIVST distribution as option for sustaining HIV service delivery amid ongoing financial uncertainty.

The study's major strength lies in its robust evaluation design, which draws from multiple data sources, allowing triangulation. Various quality assurance measures were undertaken in data collection and analysis as described in the methods section. We shared results of the study to the communities' representatives drawn from communities' study areas allowing ‘member checking’. We were able to demonstrate facilitators and barriers to successful implementation of community-level interventions, which is important for future programmes, including outside of HIV. A limitation of the study is potential reporting bias e.g. participants may have forgotten about key factors that influenced intervention success. However, this was minimized by our triangulation methods and collection of data by experienced Zimbabwean social science researchers.

## Conclusion

We found communities can successfully drive development and implementation of context-relevant HIVST distribution interventions that are acceptable and feasible to implement. Messaging on U = U can be understood by communities and is a key motivator for implementation and uptake and should therefore remain a priority and embedded within HIVST distribution at the community level. Community-driven interventions have potential to be more burdensome to some community members than others, and effort needs to be made to distribute or lessen this burden. Findings from the study can be useful for informing development of other community-led interventions, within and outside health.

## Supplementary Material

czag101_Supplementary_Data

## Data Availability

Data is available upon reasonable request to the corresponding author.

## Reflexivity statement

The majority of the authors, 10, are based in Zimbabwe where the study was conducted, while two are based in the UK, one in Switzerland, and one in South Africa. Among the authors, there are 13 females and 1 male with varying levels of research experience and seniority. More than half of the authors, nine, have significant experience researching and contributing to policy development for health system improvement. Six authors are early careers researchers in public health with experience in qualitative research. This study benefitted greatly from the diverse expertise of the authors in public health research.
