# Supplementary material for: A process evaluation of community-led distribution of HIV self-testing kits in rural communities in Zimbabwe
Source: Health Policy Plan. 2026 Aug 10;41(9):1543–55. doi: 10.1093/heapol/czag101 (PMC13678816; doi:10.1093/heapol/czag101)
Supplement: Supplementary file 1 [file czag101_supplementary_data.docx]

**SUPPLEMENTARY 1: COMMUNITY-LED OBSERVATION REPORTING TEMPLATE**

Event being observed	:

Site	:

Venue	:

Number of Villages in Cluster	:

Observer/Researcher	:

1. **Meeting 0**

**Dates:**

1. What type of venue?
2. Details of people in Attendance (e.g. leadership, men, women, young people; insert categories as necessary)

| Category | Number in attendance  Males Females | | Total | Comments |
| --- | --- | --- | --- | --- |
|  |  |  |  |  |
|  |  |  |  |  |
|  |  |  |  |  |
|  |  |  |  |  |
|  |  |  |  |  |
|  |  |  |  |  |
|  |  |  |  |  |
| Totals |  |  |  |  |
|  |  |  |  |  |

3. General attendance (is it low, moderate or High etc.)

________________________________________________________________

________________________________________________________________

4. Is this representative of the community demographic profile?

_________________________________________________________________

_________________________________________________________________

5. What are people’s reactions to the presentation on:
a) HIVST and post-test services?
b) Treatment as prevention?
c) Community-led distribution model, participation, planning and regulations *(In each case take note of interests/excitement, worries and concerns)*

6. Who actively participated in the meeting? (Leadership, men, women, young people, other - Please comment on the level of involvement of each of these groups).

7.	Were any decisions made by the community about;

a) Who the contact people would be?
b) Appointment for support visit etc.?
c) Support/incentives for distributors
d) Other

__________________________________________________________

e) How were these decisions made? (Who seem to have influenced the decisions the most?
8. Degree to which community appears to have cohesion/ or you feel there is some underlying coercion?

9. **Post Week 0 follow up**

Dates: ___________

In the observations and informal discussions with the community members	take note:

a) What is the general mood and what activities is the community engaged in?

_______________________________________________________

_______________________________________________________

b) What progress has the community made in the CLD initiative?
i. Have they met to discuss distribution strategy?

ii)	If not when do they plan to meet?

iii) What reasons are given for the delay?
c) What form of support do they require from the implementers and was this support provided?
d) Do people in this community know anything about the CLD programme? Any publicly known plans?
e) Are any CLD posters displayed in the community? Where are they mounted?

Have people seen any flyers about CLD?

f) Do members of the community look motivated to pursue the CLD cause?

__________________________________________________________

__________________________________________________________

g) Are there any worries/anxieties? About what?

__________________________________________________________

__________________________________________________________

h) What are the general views about the programme in the community? (*find a few people to ask informally*)

**Note: Visit all key public places in the villages and establish level of	awareness of the CLD?**

_____________________________________________________________________________________________________________________

10. **Week 1 Meeting – Support Visit Observations**

Dates: _______

a) Have the community decided on specific strategies to implement their community-led approach?
b) Describe the strategy in view of the following: (Who are the distributors, incentives or no incentives, safety and security of kits, confidential access to kits, procedures for detection and prevention of social harms).

i)	Who are the distributors and what was the criterion for selection?

ii)	Are they incentivised or supported in any way?

The

iii)	What are some of the distribution points e.g. dip tank, water hole, shops etc.

iv)	Storage of kits (safety and security, quality assurance)

____________________________________________________________

v)	Access to kits, equity and confidentiality

________________________________________________________________________________________________________________________c)	Procedures for prevention, detection and reporting of social harms (if any).

________________________________________________________________________________________________________________________d)	In what way does their approach meet the HTS guidelines and CLD protocol requirements?

________________________________________________________________________________________________________________________

e)	How ready is the community to move their programme forward? E.g. readiness for distributor training.

________________________________________________________________________________________________________________________

f)	Are there any programmatic issues that the community needs to re – visit or amend? Specify.

____________________________________________________________

____________________________________________________________

g)	What are the current and anticipated challenges/worries?

____________________________________________________________

____________________________________________________________

h) What form of support did they require from the implementers to move forward and was this support provided and by who?

____________________________________________________________

____________________________________________________________

i)	What are they excited about?

___________________________________________________________

___________________________________________________________

i) What are their CLD programme plans going forward?

______________________________________________________________________________________________________________________________

k)	What key decisions were made during this support meeting and what is the plan going forward?

__________________________________________________________________________________________________________________________________

11.	**Post week 1 support visit**

Dates: _________

a) Has the community met to discuss the outstanding issues?

________________________________________________________________

________________________________________________________________________________________________________________________________

b) What progress has the community made in addressing the outstanding issues?

Comment on each aspect if more than one.

_________________________________________________________________________________________________________________________________________________________________________________________________

c) Are they ready to move forward e.g. with training?

___________________________________________________________________________________________________________________________________________________________________________________________________

d) If not, what still needs to be done and, by who and by when?

____________________________________________________________________________________________________________________________________

e) What was the agreed plan going forward?
12. **Week 2 meeting**

Dates:	**___________**

For the Training of distributors report on how the training unfolded in view of the following:

| **Dates** | **Community/Cluster** | **Venue** | **Number trainees** | | **Age range** | **Comments** |
| --- | --- | --- | --- | --- | --- | --- |
|  |  |  | **Female** | **Male** |  |  |
|  |  |  |  |  |  |  |
|  |  |  |  |  |  |  |

a) How many trainees are in attendance? (Include information on venue, dates, community/cluster, and other trainee demographics).
b) Observations on topics covered, activities, participation and general understanding.

**__________________________________________________________________**

**__________________________________________________________________**

c) Is training being carried out following guidelines and content in the self-test training manual?

**__________________________________________________________________**

**__________________________________________________________________**

d) What are the participants’ reactions to the training? What seems interesting and exciting, well understood, not understood?

____________________________________________________________________________________________________________________________________

e) What is the general mood in the training environment? Are participants motivated and engaged?

__________________________________________________________________

__________________________________________________________________

f) Comment on general understanding of :
   a. Distribution procedures and guidelines.
   b. Self-testing as a method and the testing instructions and process
   c. What are the trainees excited/worried about?
g) Did training cover all key aspects as per expectation in view of CLD protocol and STAR CBDA training guidelines?

______________________________________________________________________________________________________________________________________________________________________________________________________

h) What key decisions were made, if any and by who?

__________________________________________________________________

__________________________________________________________________

i) What evidence is there to suggest that participants have achieved expected training outcomes?

______________________________________________________________________________________________________________________________________

13. **Post – training support visits/observations**

**Dates: __________**

a) Has the community started distribution?

__________________________________________________________________

__________________________________________________________________

b) Is distribution in line with guidelines for HIV testing: (age limits, voluntary	testing, and equity in access, confidentiality, linkage for confirmatory testing,	prevention, and treatment)?

c) Is distribution in line with CL Strategy with regards to;

i) Storage and safety of kits (where under what conditions)

_______________________________________________________________

________________________________________________________________

ii) Replenishments (where and when)

_______________________________________________________________

_______________________________________________________________

iii) Record keeping (What and by whom).

______________________________________________________________

______________________________________________________________

d. Is distribution compliant with the community agreed strategy? What is working/not working?

__________________________________________________________________

__________________________________________________________________

e. What is the general response to self-testing by community members? (Men, women, young people, other).

__________________________________________________

__________________________________________________

f. What is the general mood among:

i. Distributors? Any worries/concerns/excitement?

_________________________________________________________

________________________________________________________

ii. Community leaders? Any worries/concerns/excitement?

_______________________________________________________

_______________________________________________________

iii)	Members of the general community? Any	worries/concerns/excitement?

___________________________________________________

_______________________________________________________

g. What support does the community need? From who?

________________________________________________________

_______________________________________________________

h. What support has been provided? By who?

_____________________________________________________________

_____________________________________________________________

i. Any mention of social harms? (What, Who involved).

____________________________________________________________

_____________________________________________________________

14. **Community final debriefing meeting**

Dates: __________

a) **Attendance**

| Category | Number in attendance  Males Females | | Total | Comments |
| --- | --- | --- | --- | --- |
| e.g. distributors |  |  |  |  |
|  |  |  |  |  |
| Totals |  |  |  |  |

b) **Distributor details and Work Output**

| **Name** |  | **Kits received** | **Kits**  **Distributed** | **Kits left** |
| --- | --- | --- | --- | --- |
|  |  |  |  |  |
|  |  |  |  |  |

c) **Distributor experiences**
i. Dissemination of treatment as prevention message (How?	What impact)

______________________________________________

______________________________________________

ii. Acceptance of Kits (men, women, young people, other)

_____________________________________________

iii. What worked/did not work.

____________________________________________	_____________________________________________

d) **Health services provider experiences**
i. Post-test service provision (what worked/did not work)

________________________________________________

ii. Other

___________________________________________
